# No difference in laxity, proprioception and neuromuscular control after suture‐tape augmented ACL repair of acute proximal avulsions versus ACL reconstruction using hamstring autografts in young, active population

**DOI:** 10.1002/jeo2.70025

**Published:** 2024-09-26

**Authors:** Adrian Góralczyk, Paulina Zalewska, Szczepan Piszczatowski, Krzysztof Hermanowicz, Tomasz Guszczyn

**Affiliations:** ^1^ Department of Orthopaedics Humana Medica Omeda Hospital Białystok Poland; ^2^ Institute of Biomedical Engineering Bialystok University of Technology Bialystok Poland; ^3^ Ortim Orthopaedic Clinic Białystok Poland; ^4^ Department of Orthopeadics and Traumatology The Medical University of Bialystok Children's Clinical Hospital Bialystok Poland

**Keywords:** anterior cruciate ligament reconstruction, anterior cruciate ligament repair, internal bracing, neuromuscular control, proprioception, suture tape augmentation

## Abstract

**Purpose:**

The purpose of this study is to compare results of suture‐tape augmented anterior cruciate ligament (ACL) repair (internal bracing [IB]) and ACL reconstruction (ACLR) with hamstring autograft in terms of laxity, proprioception and neuromuscular control. The hypothesis was that with strict indications IB may provide better results in proprioception and neuromuscular control.

**Methods:**

Patients with unilateral ACL injury treated with IB or ACLR with hamstring autograft were enroled in this retrospective study. Anterior tibial translation (ATT) in 30° and 90° of flexion was measured with Rolimeter. The joint position sense (JPS) test was performed in 30° and 60° of flexion using Biodex System 4Pro. The time‐synchronized motion capture system and surface electromyography set were used during dynamic tasks to assess knee valgus and semitendinosus (ST) and biceps femoris (BF) activities. Comparisons between both techniques and operated versus contralateral healthy knees were performed.

**Results:**

The study groups involved 28 patients after ACLR (21.8 ± 4.8 years) and 20 patients after IB (25.8 ± 10.5 years) with the average follow‐up 30 ± 18 and 28 ± 15 months, respectively. The ATT did not differ significantly between operated groups. In 30° of flexion ATT for ACLR was significantly higher in operated than in contralateral knee (5.8 ± 2.4 mm vs. 4.3 ± 1.3 mm, *p* < 0.001). The JPS test and dynamic knee valgus presented no significant differences. The ACLR group presented significantly higher ST (*p* = 0.048) and BF (*p* = 0.012) activity comparing operated to contralateral knee in dynamic tasks.

**Conclusion:**

Suture‐tape augmented ACL repair and ACLR with hamstring autograft yield similar results in terms of laxity, proprioception and neuromuscular control.

**Level of Evidence:**

Level III: Retrospective comparative study.

**Abbreviations**
ACL: anterior cruciate ligament
ACLR: anterior cruciate ligament reconstruction
ATT: anterior tibial translation
BF: biceps femoris
IB: anterior cruciate ligament repair with suture tape augmentation (internal bracing)
EMG: electromyography
JPS test: joint position sense test
MCL: medial collateral ligament
MVC: maximum voluntary contraction
PROMs: patient‐reported outcome measures
ROM: range of motion
RTS: return to sport
ST: semitendinosus

## INTRODUCTION

Anterior cruciate ligament reconstruction (ACLR) with autograft remains the gold standard treatment option for ACL injuries, especially in young active population [11, 17]. However, the failure rate in literature is reported as 2.80%–2.84% and may come to 11.1% in a mid‐term follow‐up and up to 16.7% in a long‐term follow‐up, independently of what type of autograft had been used [5, 6, 22, 30]. Revision ACL reconstruction is a demanding procedure and surgeons have to face additional problems usually not observed in primary surgery, as bone tunnels widening, limited graft choice, concomitant intraarticular pathologies due to chronic anterior knee instability [16]. Moreover, clinical results and return to sport after revision ACL surgery are usually inferior to primary cases [16]. All presented factors have led to a revival of intensive investigations about ACL‐preserving surgeries [1, 23, 29].

Proponents of ACL primary repair emphasize that in theory, preserving native ligament allows to avoid donor‐site morbidity, restore normal knee anatomy and retain proprioceptive tailors [13, 28]. However, it is worth noting that ACL repair is a technique with limited indications and should be performed only in acute proximal ACL avulsions up to 3–6 weeks after initial trauma [26]. On the other hand, while some authors suggested that timing from injury to surgery plays no role in ACL reconstruction surgeries, current studies present that delaying ACL reconstruction for more than 12 months significantly increases the rate of medial meniscus tears [3]. According to the literature, proprioception of the ACL comprises static awareness of knee joint position, dynamic detection of knee movement and closed loop reflexes, which engages, among others, hamstring reflex responses [4]. The adequate sensation of a knee joint position being an interplay between passive (ligamentous) and active (muscles) restraints to anterior tibial translation (ATT) is extremely important in activities like jumping, pivoting and landing [4]. Thus, proprioception was proven to be of utmost importance in measuring the overall outcome of ACL tears surgical treatment [15].

Historically, the ACL repair was performed as an open surgery with an 83%–90% of clinical stability, 80% of return to sport and 79% good to excellent results [26]. However, it required incision in the joint capsule, Hoffa's fat pad and other important proprioceptive tissues, which may have been damaged, affecting final results. The development in minimal‐invasive surgery has enabled even very complicated procedures nowadays can be performed arthroscopically, avoiding the drawbacks of open procedures [7, 14]. The development of arthroscopic techniques together with the knowledge that proximal ACL tears have a good healing potential for reattachment, similar to the medial collateral ligament (MCL), led to the introduction of techniques of mechanical and biological enhancement of arthroscopic ACL repair [29]. One of currently available techniques is the suture‐tape augmented ACL repair, so called Internal Bracing® (IB) procedure (Arthrex GmBH) [12, 28]. The technique has been proven to have good to excellent clinical results enhancing the mechanical environment for native ACL healing [15, 25].

As the proponents of IB emphasize an improved proprioception is one of the most important advantages over an ACLR, the literature in this field is scarce. Therefore, the aim of our study was to compare the results of suture‐tape augmented ACL repair (IB) to results of ACL reconstruction using hamstring tendons in terms of laxity, proprioception and neuromuscular control in a young, active population. Our hypothesis was that IB may provide better results in proprioception and neuromuscular control.

## METHODS

### Participants

Patients with ACL tear treated operatively with ACL reconstruction with hamstring autograft or primary IB, who have completed at least 12 months of rehabilitation programme after the surgery, were enroled in this study. The study design was a retrospective analysis of data collected between 2019 and 2022 in the gait analysis lab. The exclusion criteria were high‐grade injury of MCL or lateral collateral ligament, injury to the posterior cruciate ligament, meniscal repair, cartilage reconstruction, history of any injury in contralateral knee, history of other surgeries involving lower extremities, and neurological and rheumatological disorders. Comparisons between both techniques as well as operated (op) and contralateral healthy knees (h) of participants were performed in terms of laxity, proprioception and neuromuscular control. The study protocol was approved by Ethical Review Board R‐I‐002/356/2017 and all participants provided written informed consent.

### Surgical treatment

Patients with ACL tear were treated arthroscopically using ACLR with hamstrings autograft or primary IB. All procedures were performed by high volume knee surgeons (K.H. and T.G.), who perform at least 300 knee surgeries per year. The primary suture‐tape augmented ACL repair was performed according to the original Internal Bracing® technique (Arthrex GmBH) [29]. The IB was performed only in patients diagnosed with proximal ACL avulsion within 3 weeks after an injury with good ACL tissue quality. When patients did not meet the inclusion criteria for IB, the ACLR with hamstrings autograft was performed instead. The semitendinosus (ST) tendon was harvested with a minimal‐invasive technique and prepared. TG performed single‐bundle ACLR, whereas KH performed both single‐bundle and double‐bundle reconstruction. In single‐bundle technique, grafts were fixed on femoral and tibial side using two TightRopes Systems (Arthrex), whereas in double‐bundle technique two TightRopes Systems (Arthrex) on femoral cortex and two cortical buttons (Medgal) on tibial side were used. After the surgery, the same rehabilitation protocol was applied in both groups. On the first postoperative day, all patients were allowed for unrestricted range of motion (ROM) and partial weight‐bearing with crutches. Quadriceps exercises set were advised. The supervised rehabilitation programme started in the second postoperative week. By the fourth postoperative week, patients were allowed for full weight‐bearing without crutches and unrestricted activities of daily living. Noncontact activities as cycling (after eighth week), running (after 5 months) and jumping (after 6 months) were gradually introduced [24]. After 9 months, all participants performed functional tests conducted by leading physiotherapists and were allowed for unrestricted sports activities.

### Study protocol

A set of biomechanical tests was performed in one session at least 12 months after surgery in the gait analysis laboratory.

ATT, as an indicator of static ACL function, was assessed with the Rolimeter (Aircast). During the examination, the patient was lying supine and fully relaxed. The ATT was measured in 30° and 90° knee flexion, three times for each angle. The values of three attempts were recorded in milimetres and the mean value was used for analysis.

**Figure 1:**
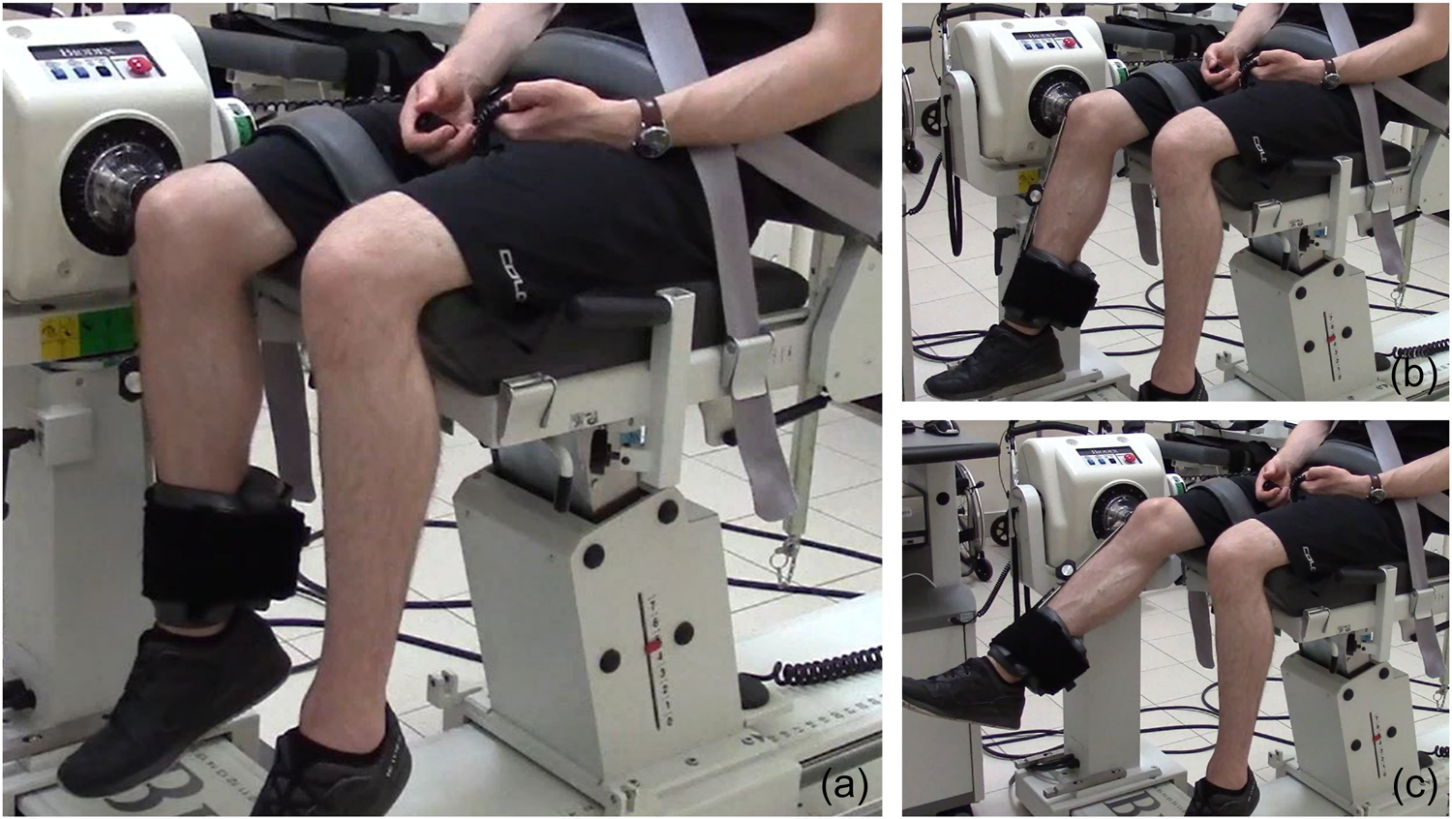
The joint position sense test performed with Biodex System 4PRO. The starting position is 95° of hip and 90° of knee flexion (a). The mean reproduction error is assessed in 60° (b) and 30° (c) of knee flexion.

The joint position sense test (JPS) was performed using the BIODEX System 4Pro (Biodex Medical Systems) according to previously reported proprioception studies [10, 21]. For the test, the patient was seated upright with the chest, pelvis and thigh of examined limb stabilized with straps (Figure 1). The starting position was 95° of hip and 90° of knee flexion. The goniometer was aligned with the lateral femoral epicondyle. The test was performed in passive–passive and active–active manner in 30° and 60° knee flexion. For the passive–passive JPS test, the knee was passively extended to set the angle, kept in position for 10 s and returned to the starting position. Next, the knee was being passively extended and the patient had to signalize with the button the position, which was previously learnt. After three training attempts, three measured and recorded attempts for each angle were performed. For the active–active JPS test, the knee was actively extended to set angle, kept in position for 10 s while the patient was instructed to contract knee periarticular muscles, and returned to starting position. Next, the patient had to actively recreate the position that was previously learnt. After three training attempts, three measured and recorded attempts for each angle were performed. The mean reproduction error of three attempts for each angle was taken into consideration during the analysis.

**Figure 2:**
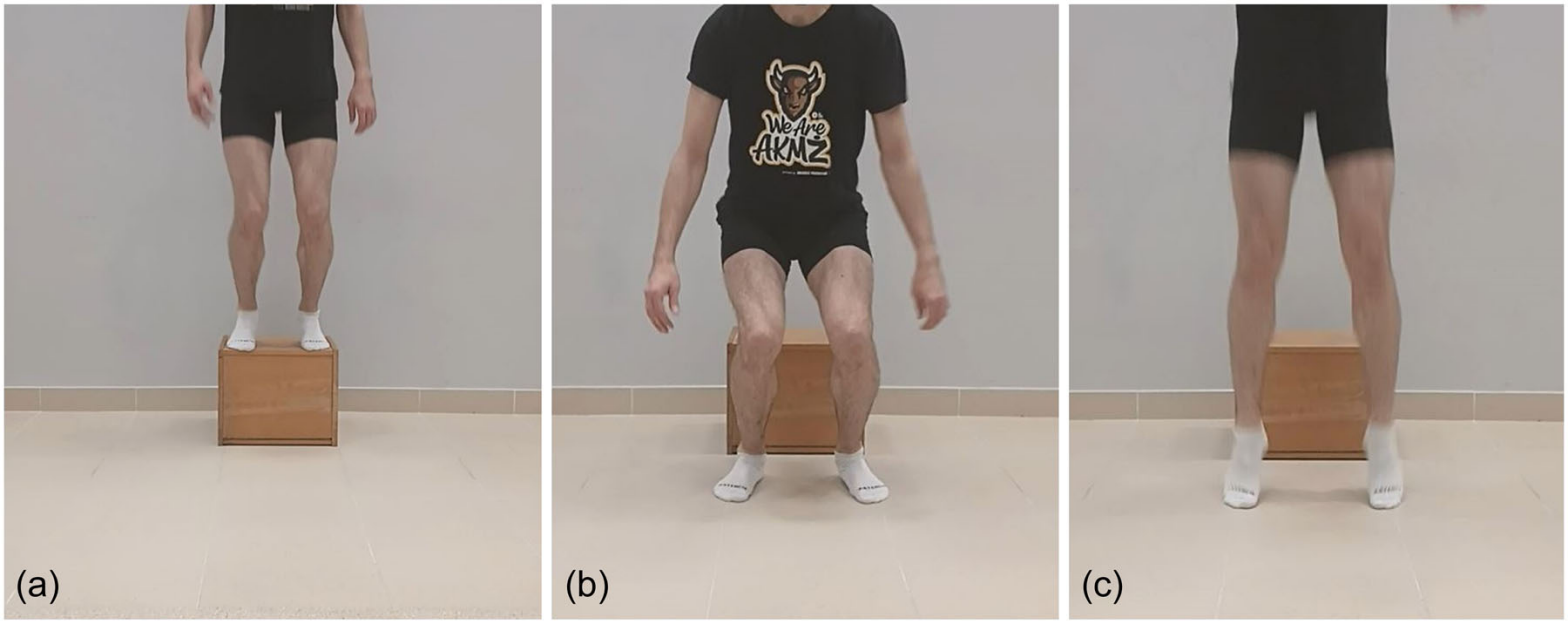
Forward landing task. Participant starts standing on 35 cm box (a) and is instructed to jump off the box, land on two feet (b) and immediately perform a vertical jump (c). Dynamic knee valgus, semitendinosus and biceps femoris activity are assessed.

Dynamic knee valgus as well as ST and biceps femoris (BF) activity were assessed during forward landing tasks. Participants started by standing on 35 cm high box and then being instructed to jump off the box, land on two feet and immediately perform a vertical jump (Figure 2). The trial was successful when participants dropped off the box without destabilization and performed a vertical jump as fast as possible without stopping. After a few practice attempts at least five proper trials were recorded. The time‐synchronized motion capture system (Qualisys AB) was used to determine dynamic knee valgus during forward landing tasks with the use of 26 passive reflective markers and four cluster‐tracking markers. Before the task, a set of four integrated SX230 electrodes (Biometrics Ltd) were placed on the participants’ skin [8, 9]. The frequency of electromyography (EMG) data collection was set at 1000 Hz and band‐pass filtered at 20–450 Hz. The activity of posterior thigh muscles during forward landing tasks for operated and contralateral limb was determined. To normalize the EMG results, the maximum voluntary contraction (MVC) procedure was performed [8]. To obtain meaningful amplitude values from the raw EMG data the RMS 150 (root mean square, *t* = 150 ms) procedure was used in the Biometrics DataLog programme. ST and BF activity were determined for the first (0%) and the last (100%) contact of feet with the ground and for the moment of the highest knee flexion angle (MAX). Muscle activity was normalized as %MVC. Kinematic data were processed in Visual 3D (C‐Motion) for the same time moments.

### Statistical analysis

A posthoc power analysis was conducted to determine the required sample size, aimed at ensuring sufficient statistical power to detect a significant difference between two independent sample means, as well as within a paired design. The effect size, Cohen's *d*, was assumed to be *d* = 0.65, fitting within the medium range (0.5–0.8) as defined by Cohen's conventions. The analysis utilized a two‐sided hypothesis test with a significance level *α* = 0.05. The desired power (1 − *β*) was established at 0.80, reflecting an 80% probability of correctly rejecting a false null hypothesis. To achieve this level of power, at least 39 participants per group and 21 pairs of observers were required for the between‐group and within‐group design. The significance threshold for this analysis was set at *α* = 0.05. To assess the normality of numerical variables, the Shapiro–Wilk test was employed. Numerical variables were described using mean (*M*) and SD were presented in terms of count and percentage within each category. The differences between two independent groups were assessed using Welch's *t* test, which accounts for variances that are not assumed to be equal across groups. For dependent measures, differences between means were evaluated using a paired *t* test. In terms of effect size, Cohen's *g* was calculated. Medium effect sizes ranging from 0.5 to 0.8 suggested the potential for clinical significance, prompting the recommendation for additional research with a larger sample size to verify these findings. Conversely, Cohen's *g* values exceeding 0.8 were considered demonstrative of a clinical effect.

The analyses were conducted using the R Statistical language (version 4.3.1; R Core Team, 2023) on Windows 10 ×64 (build 19045).

## RESULTS

The study groups involved 28 patients after ACLR (10 female/18 male; 21.8 ± 4.8 years) and 20 patients after IB procedure (eight female, 12 male; 25.8 ± 10.5 years) with the average follow‐up 30 ± 18 months and 28 ± 15 months, respectively. Anthropometric data are summarized in Table 1.

**Table 1:** Anthropometric characteristic of study groups.

Group parameter	IB (*n* = 20)	ACLR (*n* = 28)	*p*
	8 F/12 M	10 F/18 M	
Age (years)	25.0 ± 10.5	21.8 ± 4.8	0.161 (*g* = 0.390)
Height (m)	1.76 ± 0.11	1.74 ± 0.12	0.661 (*g* = 0.130)
Mass (kg)	84.0 ± 14.5	76.8 ± 16.2	0.241(*g* = 0.340)
BMI (kg/m^2^)	26.8 ± 5.2	25.3 ± 4.4	0.272 (*g* = 0.330)
Time postsurgery (months)	28 ± 15	30 ± 18	0.731 (*g* = 0.100)
Thigh circumference (cm)	48.2 ± 5.8 op 48.4 ± 6.0 h	46,8 ± 4.9 op 47.6 ± 5.2 h	0.971 (ACLR op vs. ACLR h) 0.999 (IB op vs. IB h) 0.876 (ACLR op vs. IB op) 0.972 (ACLR h vs. IB h)

Abbreviations: ACLR, anterior cruciate ligament reconstruction with hamstring autograft; ACLR h, contralateral healthy knee of patient after unilateral ACL reconstruction; ACLR op, knee after ACL reconstruction; F, female; *g*, Cohen's g value; IB, anterior cruciate ligament repair with suture tape augmentation; IB h, contralateral healthy knee of patient after unilateral ACL repair with suture tape augmentation; IB op, knee after ACL repair with suture tape augmentation; M, male.

Static ATT in 30° in operated knees was measured as 5.8 ± 3.1 mm for IB and 5.8 ± 2.4 mm for ACLR group, and did not differ significantly (*p* = 0.981, *g* = −0.010). In 90° knee flexion, the ATT was measured as 4.8 ± 3.4 mm for operated knee in patients after IB procedure and 4.2 ± 2.0 mm for operated knee in patients after ACLR, and also did not differ significantly (*p* = 0.432, *g* = −0.024). In 30° knee flexion, ATT for ACLR group was significantly higher in operated (5.8 ± 2.4 mm) than in contralateral knee (4.3 ± 1.3 mm) (*p* < 0.001, *g* = 0.820). Table 2 summarizes the results of ATT measurements.

**Table 2:** ATT measured with rolimeter.

Group parameter	30°		90°	
	Mean ± SD (mm)	Side‐to‐side difference (mm) with CI 95%	Mean ± SD (mm)	Side‐to‐side difference (mm) with CI 95%
IB op	5.8 ± 3.1	0.6 (−0.1, 1.3)	4.8 ± 3.4	0.7 (−0.03, 1.49)
IB h	5.2 ± 2.9		4.1 ± 2.7	
ACLR op	5.8 ± 2.4	1.5 (0.8, 2.2)	4.2 ± 2.0	0.1 (−0.22, 0.50)
ACLR h	4.3 ± 1.3		4.1 ± 1.9	
*p*	**<0.001 (ACLR op vs. ACLR h) (*g*** = **0.820)** 0.083 (IB op vs. IB h) (*g* = 0.039) 0.981 (ACLR op vs. IB op) (*g* = −0.010) 0.195 (ACLR h vs. IB h) (*g* = −0.400)		0.424 (ACLR op vs. ACLR h) (*g* = 0.150) 0.058 (IB op vs. IB h) (*g* = 0.430) 0.432 (ACLR op vs. IB op) (*g* = −0.0240) 0.907 (ACLR h vs. IB h) (*g* = −0.030)	

*Note*: The values which revealed statistically significant differences are bolded in the table.

Abbreviations: ACLR, anterior cruciate ligament reconstruction with hamstring autograft; ACLR h, contralateral healthy knee of patient after unilateral ACL reconstruction; ACLR op, knee after ACL reconstruction; ATT, anterior tibial translation; CI, confidence interval; *g*, Cohen's *g* value; IB, anterior cruciate ligament repair with suture tape augmentation; IB h, contralateral healthy knee of patient after unilateral ACL repair with suture tape augmentation; IB op, knee after ACL repair with suture tape augmentation.

No statistically significant differences were observed between ACLR and IB groups in the mean reproduction error in a passive and active JPS test in both 30° and 60° knee flexion. In 60° knee flexion in active‐active JPS test statistically significant difference was observed in mean reproduction error between IB op and IB h groups (*p* = 0.027, *g* = 0.550). Periarticular muscle contraction in active JPS test in 30° improves significantly the mean reproduction error forcontralateral, healthy knee in ACLR (*p* = 0.003, *g* = 0.620) group, but did not affect the results in operated knees. No statistically significant differences were observed among other groups. All results of the JPS test are presented in Tables 3 and 4.

**Table 3:** Mean reproduction error in passive and active JPS test in 30°.

Group	30° passive	30° active	*p*
parameter	(°)	(°)	(passive vs. active)
IB op	6.3 ± 3.6	4.4 ± 2.3	0.109 (*g* = 0.370)
IB h	6.3 ± 2.9	4.3 ± 3.1	0.091 (*g* = 0.390)
ACLR op	5.3 ± 2.9	4.7 ± 2.9	0.370 (*g* = 0.170)
ACLR h	5.0 ± 2.8	3.3 ± 1.7	**0.003 (*g*** = **0.620)**
*p* (between groups)	0.371 (ACLR op vs. ACLR h) (*g* = 0.170) 0.965 (IB op vs. IB h) (*g* = 0.010) 0.350 (ACLR op vs. IB op) (*g* = −0.280) 0.133 (ACLR h vs. IB h) (*g* = −0.045)	**0.016 (ACLR op vs. ACLR h) (*g*** = **0.470)** 0.898 (IB op vs. IB h) (*g* = 0.030) 0.383 (ACLR op vs. IB op) (*g* = 0.110) 0.230 (ACLR h vs. IB h) (*g* = −0.037)	

*Note*: The values which revealed statistically significant differences are bolded in the table.

Abbreviations: ACLR, anterior cruciate ligament reconstruction with hamstring autograft; ACLR h, contralateral healthy knee of patient after unilateral ACL reconstruction; ACLR op, knee after ACL reconstruction; ATT, anterior tibial translation; CI, confidence interval; *g*, Cohen's *g* value; IB, anterior cruciate ligament repair with suture tape augmentation; IB h, contralateral healthy knee of patient after unilateral ACL repair with suture tape augmentation; IB op, knee after ACL repair with suture tape augmentation, JPS, joint position sense.

**Table 4:** Mean reproduction error in passive and active JPS test in 60°.

Group parameter	60° passive	60° active	*p*
	(°)	(°)	(passive vs. active)
IB op	3.4 ± 1.7	4.8 ± 2.8	0.061 (*g* = −.045)
IB h	2.9 ± 1.4	3.9 ± 3.0	0.200 (*g* = −0.290)
ACLR op	3.5 ± 1.6	3.9 ± 2.0	0.444 (−0.140)
ACLR h	2.8 ± 2.1	3.3 ± 2.0	0.299 (*g* = −0.190)
*p* (between groups)	0.117 (ACLR op vs. ACLR h) (*g* = 0.300) 0.262 (IB op vs. IB h) (*g* = 0.250) 0.832 (ACLR op vs. IB op) (*g* = 0.060) 0.870 (ACLR h vs. IB h)(*g* = −0.050)	0.084 (ACLR op vs. ACLR h) (*g* = 0.330) **0.027 (IB op vs. IB h) (*g*** = **0.550)** 0.110 (ACLR op vs. IB op) (*g* = −0.500) 0.444 (ACLR h vs. IB h)(*g* = −0.023)	

*Note*: The values which revealed statistically significant differences are bolded in the table.

Abbreviations: ACLR, anterior cruciate ligament reconstruction with hamstring autograft; ACLR h, contralateral healthy knee of patient after unilateral ACL reconstruction; ACLR op, knee after ACL reconstruction; *g*, Cohen's *g* value; IB, anterior cruciate ligament repair with suture tape augmentation; IB h, contralateral healthy knee of patient after unilateral ACL repair with suture tape augmentation; IB op, knee after ACL with suture tape augmentation; JPS, joint position sense.

Dynamic knee valgus angle measured during forward landing task revealed no statistically significant differences between the groups. For IB group, the knee valgus angle was measured as 0.02 ± 4.8° during the first landing, −4.5 ± 9.6° in maximal knee flexion and −2.4 ± 3.3° during take‐off for operated knee and −0.1 ± 4.5°, −3.0 ± 7.4° and −1.6 ± 3.3° for contralateral knee, respectively. For ACLR group, the knee valgus angle was measured as 1.9 ± 5.5° during the first landing, −1.4 ± 9.2° in maximal knee flexion and −0.3 ± 4.9° during take‐off for operated knee, and −0.9 ± 5.3°, −2.9 ± 9.8° and −0.04 ± 5.1° for contralateral knee, respectively.

Muscle activity, measured as %MVC, was significantly higher for BF in patients after ACL reconstruction in operated limb in comparison to contralateral limb in first feet‐floor contact moment (25.7 ± 11.4 vs. 19.8 ± 10.5, *p* < 0.001, *g* = 0.800) and during take‐off (49.9 ± 16.6 vs. 40.3 ± 12.6, *p* = 0.012, *g* = 0.540), as well as for ST during take‐off (46.0 ± 20.3 vs. 33.7 ± 18.3, *p* = 0.049, *g* = 0.460). There were no statistically significant differences observed between other groups. Tables 5 and 6 summarize the results of posterior thigh muscle activity analysis in dynamic task.

**Table 5:** The activity of BF measured with surface EMG during forward landing task.

Parameter	IB	ACLR	*p*
0% (%MVC)	20.3 ± 11.5 op 20.4 ± 14.1 h	25.7 ± 11.4 op 19.8 ± 10.5 h	**<0.001 (ACLR op vs. ACLR h) (*g*** = **0.800)** 0.845 (IB op vs. IB h) (*g* = 0.050) 0.141 (ACLR op vs. IB op) (*g* = 0.470) 0.872 (ACLR h vs. IB h) (*g* = 0.050)
Max (%MVC)	19.7 ± 12.2 op 23.5 ± 17.4 h	25.9 ± 13.5 op 24.1 ± 15.1 h	0.542 (ACLR op vs. ACLR h) (*g* = 0.130) 0.455 (IB op vs. IB h) (*g* = 0.180) 0.146 (ACLR op vs. IB op) (*g* = 0.470) 0.907 (ACLR h vs. IB h) (*g* = 0.040)
100% (%MVC)	41.2 ± 14.7 op 38.2 ± 16.3 h	49.9 ± 16.6 op 40.3 ± 12.6 h	**0.012 (ACLR op vs. ACLR h) (*g*** = **0.540)** 0.537 (IB op vs. IB h) (*g* = 0.120) 0.086 (ACLR op vs. IB op) (*g* = 0.540) 0.658 (ACLR h vs. IB h) (*g* = 0.140)

*Note*: The values which revealed statistically significant differences are bolded in the table.

Abbreviations: ACLR, anterior cruciate ligament reconstruction with hamstring autograft; ACLR h, contralateral healthy knee of patient after unilateral ACL reconstruction; ACLR op, knee after ACL reconstruction; BF, biceps femoris; EMG, electromyography; *g*, Cohen's *g* value; IB, anterior cruciate ligament repair with suture tape augmentation; IB h, contralateral healthy knee of patient after unilateral ACL repair with suture tape augmentation; IB op, knee after ACL with suture tape augmentation; JPS, joint position sense; MVC, maximum voluntary contraction.

**Table 6:** The activity of ST measured with surface EMG during forward landing task.

Parameter	IB	ACLR	*p*
0% (%MVC)	28.4 ± 14.6 op 24.7 ± 10.8 h	33.8 ± 20.3 op 26.8 ± 19.6 h	0.431 (ACLR op vs. ACLR h) (*g* = 0.180) 0.369 (IB op vs. IB h) (*g* = 0.230) 0.366 (ACLR op vs. IB op) (*g* = 0.300) 0.697 (ACLR h vs. IB h) (*g* = 0.130)
Max (%MVC)	26.3 ± 11.9 op 20.8 ± 13.4 h	29.1 ± 18.1 op 24.2 ± 17.6 h	0.595 (ACLR op vs. ACLR h) (*g* = 0.130) 0.087 (IB op vs. IB h) (*g* = 0.480) 0.594 (ACLR op vs. IB op) (*g* = 0.180) 0.548 (ACLR h vs. IB h) (*g* = 0.210)
100% (%MVC)	40.3 ± 25.9 op 35.9 ± 20.8 h	46.0 ± 20.3 op 33.7 ± 18.3 h	**0.049 (ACLR op vs. ACLR h) (*g*** = **0.460)** 0.472 (IB op vs. IB h) (*g* = 0.190) 0.492 (ACLR op vs. IB op) (*g* = 0.240) 0.725 (ACLR h vs. IB h) (*g* = −0.110)

*Note*: The values which revealed statistically significant differences are bolded in the table.

Abbreviations: 0%, the moment of first feet‐floor contact; 100%, the moment of take‐off; ACLR, anterior cruciate ligament reconstruction with hamstrings autograft; ACLR h, contralateral healthy knee of patient after unilateral ACL reconstruction; ACLR op, knee after ACL reconstruction; BF, biceps femoris; EMG, electromyography; *g*, Cohen's *g* value; IB, anterior cruciate ligament repair with suture tape augmentation; IB h, contralateral healthy knee of patient after unilateral ACL repair with suture tape augmentation; IB op, knee after ACL repair with suture tape augmentation; Max, the moment of maximum knee flexion; MVC, maximum voluntary contraction; ST, semitendinosus.

## DISCUSSION

The most important finding of our study is that in a direct comparison, suture‐tape augmented ACL repair and ACLR with hamstring autograft yield similar results in terms of laxity, proprioception and neuromuscular control. However, assuming that contralateral limb reflects the natural status for particular individual, IB appears to restore native knee laxity and neuromuscular control better compared to ACLR in young active population.

More and more data in literature suggests that IB may provide similar or even better clinical results than gold‐standard ACLR with hamstring autograft [15, 25, 27]. Whereas most studies focused on patient‐reported outcomes measures (PROMs), knee ROM, stability and return to sport (RTS), there is few studies on proprioception and neuromuscular control, which were highlighted by proponents of IB as the major potential advantages over the ACLR. Bühl et al. [2] in their study about functional leg performance 2 years after ACL surgery compared IB group with ACLR with hamstring autograft and found no statistically significant differences in operated legs between groups, but did find moderate difference in IB group between operated and nonoperated legs in hop tests performance and moderate difference in ACLR group between operated and nonoperated legs in proprioception. The same group of researchers in another study revealed that IB and ACLR groups did not differ significantly in term of PROMs, ATT and absolute and normalized isokinetic muscle strength, but both groups reached inferior results in comparison to healthy control [18]. In their studies the mean patients age was 36.8 years for ACL‐IB, 37.0 years for ACLR and 37.0 years for controls. In contrast, we enroled younger patients with the average age of 25.8 ± 10.5 years for IB group and 21.8 ± 4.8 years for ACLR group (*p* = 0.161), which better reflects the most commonly affected patient population [19]. In our study, we focused on ATT, JPS, dynamic knee valgus and muscle activity in dynamic tasks in patients after IB and ACLR with hamstring autograft. In contrast to the study of Szwedowski et al. [25], we did not observe any statistically significant differences in ATT between IB and ACLR group, but we revealed significant differences within ACLR group between operated and non‐operated leg in 30° knee flexion, which is a functional position for the ACL during sports activities. None of the studied groups achieved a 3 mm side‐to‐side difference in ATT between operated and contralateral healthy knees in any knee flexion angles, which is considered as clinically important joint laxity [4]. In contrast to the results of Bühl et al. [2], we did not find statistically significant differences in JPS test between IB and ACLR, and within groups in 30° and 60° knee flexion. Interestingly, periarticular muscle contraction in active JPS test significantly affected results diminishing the mean reproduction error for ACLR contralateral limb. It has been proven that proprioceptive disfunction after ACLR with autografts may persist even after RTS, even in a high‐level athletes and that contralateral knee remains at higher risk of ACL tear [20]_._

An interesting finding of our study is that during a dynamic task we observed a significantly higher posterior thigh muscles activity in operated leg in comparison to contralateral limb in ACLR group in a first feet‐ground contact moment and during a take‐off. BF and ST work synergistically to ACL dynamically preventing ATT. Thus, our finding may be explained as agonistic muscles overactivation due to persistent ACL laxity after reconstruction, what is rather unlikely due to good results in ATT testing with a Rolimeter. The other potential explanations may be closed loop reflexes disorders due to injury of a native ACL and substitute with a graft, or even muscle protection of the reconstructed ACL learnt in a rehabilitation process. On the other hand, this overactivity may be explained also by the fact that IB was performed in the first 3 weeks after injury, whereas the time from injury to surgery for ACLR group was longer. This period may be responsible for development of muscle overactivity as the tool for knee protection against instability. This phenomenon needs to be further investigated.

The undoubted strength of our study is the population which we enroled, which reflects the typical patients with ACL injuries requiring surgical treatment. Moreover, we tried to assess each component of the ACL proprioception: static stability, dynamic detection of knee movement and awareness of knee joint position, closed loop reflex involving synergistic muscles response [4]. However, there were also some limitations. The most important is a small sample size due to strict inclusion criteria. Because the sample size was smaller than determined in posthoc power analysis, we were forced to introduce Cohen's *g* value to assess the effect size. The time of surgery and the pattern of ACL injury were not homogenous in both groups due to specific indication for each technique, especially in case of IB. Furthermore, a complicated study protocol in biomechanical laboratory required a long and precise explanation of the task to the participants, a good compliance and a few trial attempts before proper measurements. Finally, statistically significant differences, which we observed in ATT, BF and ST activity in ACLR group comparing operated and contralateral limbs, may not exceed minimal clinically important difference, which is not possible to determine in our study due to its pure biomechanical character.

## CONCLUSION

Primary suture‐tape augmented ACL repair and ACLR with hamstring autograft yield similar results in terms of laxity, proprioception and neuromuscular control in a population of young, active patients. In comparison to contralateral, healthy knees, the IB appears to have superior results over the ACLR in restoring native parameters of knee stability and neuromuscular control, but no proprioception.

## AUTHOR CONTRIBUTIONS

All authors contributed to the study conception and design. Material preparation, data collection and analysis were performed by Paulina Zalewska, Adrian Góralczyk and Szczepan Piszczatowski. The first draft of the manuscript was written by Adrian Góralczyk and all authors commented on previous versions of the manuscript. All authors read and approved the final manuscript.

## CONFLICT OF INTEREST STATEMENT

The authors declare no conflict of interest.

## ETHICS STATEMENT

The study was approved by Ethical committee approval number: R‐I‐002/356/2017. All patients provided written consent before participation in the study.

## Data Availability

We state all the data are available on demand.
